# Systematic review and meta-analysis of the proportion of non-typhoidal *Salmonella* cases that develop chronic sequelae

**DOI:** 10.1017/S0950268814002829

**Published:** 2014-10-30

**Authors:** J. KEITHLIN, J. M. SARGEANT, M. K. THOMAS, A. FAZIL

**Affiliations:** 1 Centre for Public Health and Zoonoses, University of Guelph, Guelph, Ontario, Canada; 2 Department of Population Medicine, Ontario Veterinary College, Guelph, Ontario, Canada; 3 Centre for Food-borne, Environmental and Zoonotic Infectious Diseases, Public Health Agency of Canada, Guelph, Ontario, Canada; 4 Laboratory for Foodborne Zoonoses, Public Health Agency of Canada, Guelph, Ontario, Canada

**Keywords:** Inflammatory bowel disease, reactive arthritis, salmonellosis

## Abstract

The objective of this systematic review and meta-analysis was to estimate the proportion of cases of non-typhoidal salmonellosis (NTS) that develop chronic sequelae, and to investigate factors associated with heterogeneity. Articles published in English prior to July 2011 were identified by searching PubMed, Agricola, CabDirect, and Food Safety and Technology Abstracts. Observational studies reporting the number of NTS cases that developed reactive arthritis (ReA), Reiter's syndrome (RS), haemolytic uraemic syndrome (HUS), irritable bowel syndrome (IBS), inflammatory bowel disease (IBD) or Guillain–Barré syndrome (GBS), Miller–Fisher syndrome (MFS) were included. Meta-analysis was performed using random effects and heterogeneity was assessed using the *I*^2^ value. Meta-regression was used to explore the influence of study-level variables on heterogeneity. A total of 32 studies were identified; 25 reported on ReA, five reported on RS, seven reported on IBS, two reported on IBD, two reported on GBS, one reported on MFS, and two reported on HUS. There was insufficient data in the literature to calculate a pooled estimate for RS, HUS, IBD, GBS, or MFS. The pooled estimate of the proportion of cases of NTS that developed ReA and IBS had substantive heterogeneity, limiting the applicability of a single estimate. Thus, these estimates should be interpreted with caution and reasons for the high heterogeneity should be further explored.

## INTRODUCTION

Non-typhoidal *Salmonella* is an important foodborne pathogen, with an estimated 93·8 million cases and about 155 000 deaths globally per year [1]. Common sources of infection include contaminated food, such as meat, eggs and produce [2, 3] and via the faecal–oral route after contact with infected animals [4]. Acute symptoms associated with salmonellosis include diarrhoea, fever, headache and abdominal pain. Salmonellosis has also been implicated in the development of chronic sequelae such as reactive arthritis (ReA) [5] and irritable bowel syndrome [6].

Burden of disease (BOD) estimates can be used by researchers and policy makers to help prioritize funding and identify intervention opportunities. Efforts have been made by many countries to estimate the BOD associated with foodborne diseases such as *Salmonella* (for examples, see [1, 7–9]). There is variability in the sequelae included in these estimates and in the sources of data for estimating the frequency of the sequelae [10].

Systematic review and meta-analysis provide an opportunity to assess all of the available literature on a topic in a transparent and reproducible manner, by identifying all of the literature on a topic and combining the results across studies [11]. The purpose of this systematic review and meta-analysis was to estimate the proportion of cases of non-typhoidal salmonellosis (NTS) that develop ReA or Reiter's syndrome (RS), Guillain–Barré syndrome (GBS), Miller Fisher syndrome (MFS), irritable bowel syndrome (IBS), haemolytic uraemic syndrome (HUS), inflammatory bowel disease (IBD) including ulcerative colitis (UC), and Crohn's disease (Crohn's) and to use meta-regression to explore the study-level variables that contributed to variation in estimates. The review was part of a larger project that also estimated the proportion of individuals with *E. coli* O157 and *Campylobacter* who developed chronic sequelae [12, 13].

## MATERIALS AND METHODS

### Literature search and inclusion-exclusion criteria/data variables

The literature search was conducted to identify chronic sequelae for multiple foodborne pathogens. The following search terms were entered into four electronic databases (Medline via PubMed, Agricola, CabDirect, and Food Safety and Technology Abstracts); (‘*Escherichia coli* O157’, or, ‘O157’, ‘VTEC’, ‘STEC’, ‘O157:H7’ or *Salmonella* or *Campylobacter*) and (‘sequel*’, ‘long-term’, ‘long term’, ‘chronic’, ‘Guillain*’, ‘HUS’, ‘hemolytic uremic syndrome’, ‘haemolytic uraemic syndrome’, ‘hemorrhagic uremic syndrome’, ‘haemorrhagic uraemic syndrome’, ‘Reiter*’, ‘complication*’, ‘arthritis’, ‘irritable bowel syndrome’, ‘IBS’, ‘post infectious irritable bowel syndrome’ or ‘inflammatory bowel disease’), without language restrictions to identify citations from prior to July 2011. The reference lists of relevant studies were reviewed to identify additional studies not located by the initial search. This paper presents results for *Salmonella* and the chronic sequelae of ReA including RS, IBS, IBD including UC and Crohn's, GBS including MFS, and HUS.

Three levels of screening for eligibility were performed. The first level of screening was performed by a single reviewer using titles and abstracts and excluded references that did not include information on either *Campylobacter, E. coli* O157 or non-typhoidal *Salmonella*, did not discuss chronic sequelae in humans or were laboratory-based studies, randomized control trials or reviews. The second level of screening, also based on titles and abstracts, was performed by two independent reviewers with conflicts resolved by consensus. This level restricted results to specific pathogen types (excluding *Salmonella typhi* and *S. paratyphi*) and the specific chronic sequelae of interest. The final round of screening was performed using the full-text publications and identified studies with the information required to estimate the proportion of NTS cases that developed a chronic sequelae. Those publications that presented the opposite relationship, the number of sequelae cases with evidence of past *Salmonella* exposure, were excluded.

Full publications that met all eligibility criteria then underwent data extraction. This was performed by two independent reviewers extracting data from each article. Conflicts were resolved via consensus with input from a third reviewer where required. Data were extracted on population (year, country, age range and gender distribution for *Salmonella* cases), *Salmonella* species or serotype, study directionality (retrospective *vs*. prospective), source of data (surveillance *vs*. outbreak *vs*. hospitalized cases), season and decade of data collection, if sequelae cases were disease negative prior to salmonellosis, categories describing both the *Salmonella* diagnosis and sequelae diagnosis, the length of time between *Salmonella* diagnosis and sequelae diagnosis (follow-up time) and outcomes (number of cases of *Salmonella*, number of cases of NTS who developed a chronic sequelae). Prospective studies were those where cases of NTS were identified and the assessment for sequelae occurred at a time point in the future. Retrospective studies were those where both the identification as a case of NTS and sequelae diagnosis had already occurred prior to data collection for the study. Diagnosis of *Salmonella* was categorized as confirmed or probable based on the description of diagnostic methods provided in each publication. Confirmed NTS cases were those where cases were identified by culture, serology or DNA-based tests and probable cases were those identified as a case based on the clinical case definition given in the study. These included self-reported illness from survey data and cases without specific diagnostic test results that were linked to outbreaks. Diagnoses of the sequelae were categorized as assessment by specialist, physician diagnosed/taken from medical records, self-reported based on validated scale or self-reported.

The outcome of interest for this systematic review was the proportion of NTS cases that developed a chronic sequela. As some publications reported multiple methods of diagnosing both *Salmonella* (e.g. a study may have reported both probable and confirmed cases) and the sequelae (e.g. a study may have reported both self-reported and specialist confirmed cases of the sequelae), as well as multiple data sources (e.g. both outbreak-associated and hospitalized cases), it was possible for multiple estimates to be reported from the same study for the proportion of NTS cases that developed a sequela. Therefore the term ‘outcome’ was used to describe the probability of a case of NTS developing a chronic sequela for each of these various combinations.

### Statistical analysis

For each outcome, the proportion of NTS cases that developed specific chronic sequelae was calculated as the number of persons developing a sequela divided by the total number of NTS cases. Standard errors and confidence intervals for a single proportion were derived. Prior to analysis, to incorporate the influence of study size on the outcome, adjusted proportions and standard errors were calculated using a logit transformation [14].

Meta-analysis was performed for sequelae with more than 10 outcomes using a random-effects model and the DerSimonian & Laird method to derive the summary estimate [11]. Heterogeneity was assessed using the *I*^2^ value [15]. To allow for inclusion in the meta-analysis, a count of 0·5 was added or subtracted from the number of sequelae cases in those studies reporting a chronic sequelae outcome of 0% or 100%, respectively [16]. Meta-regression was used to explore potential sources of heterogeneity if the *I*^2^ value was higher than 25% and if more than ten outcomes were present for the sequelae of interest. The source of data (outbreak *vs*. surveillance *vs*. hospitalized cases), the method of diagnosing *Salmonella, Salmonella* serotype, the method used to diagnose the sequelae, disease status prior to illness with *Salmonella*, country, study directionality, study decade, season, group size and follow-up time were considered as explanatory variables in the meta-regression. All statistical analyses were performed in Stata version 12 (StataCorp, USA).

Categorical variables representing season, decade of study, group size (number of cases of NTS) and follow-up time (time from diagnosis with *Salmonella* to sequela diagnosis) were generated from the data provided in the studies for use in meta-regression. In the northern hemisphere season was categorized as autumn (September–November), winter (December–February), spring (March–May) and summer (June–August). In the southern hemisphere season was classified as autumn (March–May), winter (June–August), spring (September–November) and summer (December–February). Decade of study was classified based on the decade when data collection began. Group size was divided into small (*n* ⩽ 100), medium (*n* = 101–1000), large (*n* = 1001–10 000), and extra-large (*n* = > 10 000). Due to the transient nature and varying duration of symptoms associated with many sequelae, follow-up time was divided into three categories; <3 months (90 days), >90 days to <1 year (365 days) and ⩾1 year (365 days).

Using meta-regression, significance was determined first by univariable analysis (*P* ⩽ 0·05 was considered significant), then significant variables were included in a backwards multivariable model. Meta-regression was performed using logit-transformed outcomes and logit-transformed within-study standard errors. Those variables that remained significant (*P* ⩽ 0·05) were further explored with subgroup meta-analysis if there were at least two outcomes available in the data for each level of the explanatory variable.

### Assessment of factors associated with internal/external validity

A pre-established risk of bias assessment was not conducted. Alternatively, data were extracted on whether or not information on the following variables was reported in the publication; study directionality (retrospective *vs*. prospective), source of data (outbreak *vs*. surveillance *vs*. hospitalized cases), method of diagnosis for both *Salmonella* and for the sequelae, follow-up time, whether sequelae cases were disease-negative prior to salmonellosis, and population information (country, gender distribution, age range). The definition for sequelae diagnosis was divided into two components; the method of diagnosis (physician diagnosed *vs*. self-reported) and if specific diagnostic criteria were provided.

## RESULTS

### Systematic review

#### Study selection

**Fig. 1.**
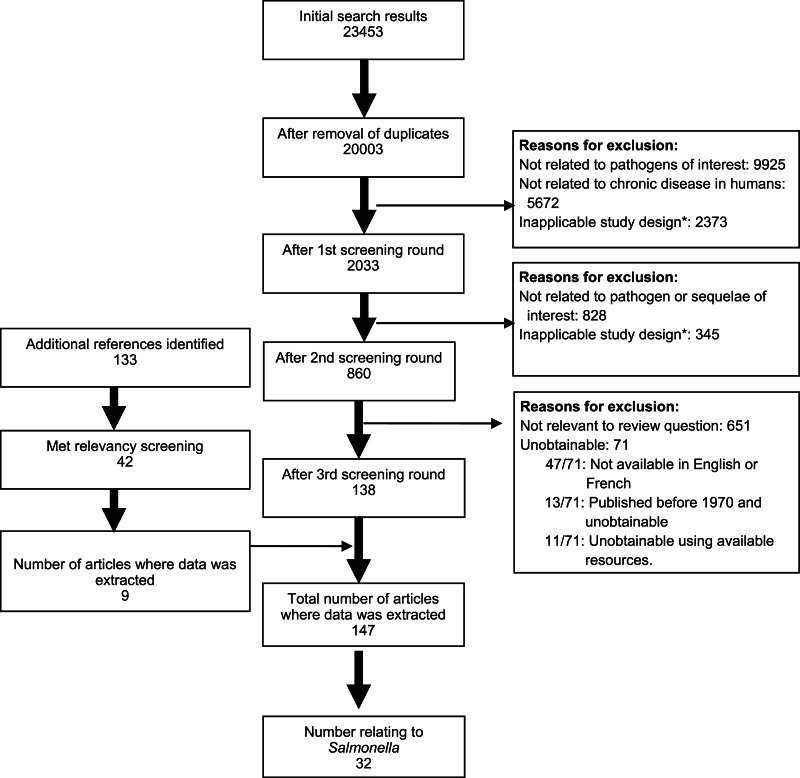
Flow chart of results from systematic review for *Salmonella* and chronic sequelae. * Excluded study designs were randomized control trials, laboratory-based studies and those that selected subjects based on sequelae and determined previous *Salmonella* exposure.

The results of the systematic literature search are summarized in Figure 1. After screening and identification of relevant articles from reference lists there were 147 publications that met the inclusion criteria of which 32 contained information on *Salmonella* and chronic sequelae. Most publications reported on a single sequela, although the number of sequelae reported ranged from 1 to 6 (Table 1).

**Table 1.** Population characteristics for studies relating to chronic sequelae of *Salmonella* published before July 2011

First author, year [ref.]	Sequelae	Country	Age range* (of *Salmonella* cases)	% Female (for *Salmonella* cases)	Source of data	Study directionality	Outbreak source	Date for data collection	Season
Ternhag, 2008 [21]	ReA, HUS, IBS, UC, Crohn's	Sweden	All ages	51	Surveillance†	Retrospective	n.a.	1997–2004	All
Doorduyn, 2008 [22]	ReA, Reiter's, GBS, MFS, IBS, IBD	Netherlands	n.r.	n.r.	Surveillance	Prospective	n.a.	2002–2003, 2005	All
Ekman, 2000 [23]	ReA	Finland	Adults	53	Surveillance	Prospective	n.a.	1998–1999	All
Schiellerup, 2008 [24]	ReA	Denmark	Adults	57·1	Surveillance	Prospective	n.a.	2002–2003	All
Townes, 2008 [25]	ReA	USA	All ages	50	Surveillance	Prospective	n.a.	2002–2004	All
Jess, 2011 [26]	Crohn's, UC	Denmark	All ages	50	Surveillance	Retrospective	n.a.	1992–2008	All
Buxton, 2002 [27]	ReA	Can.a.da	All ages	53	Surveillance	Prospective	n.a.	1999–2000	All
Arnedo-Pena, 2010 [28]	ReA	Spain	n.r.	50	Outbreak in community	Prospective	Food: meat	2005	Summer
Cowden, 1989 [29]	UC	England	All ages	46	Outbreak in community	Prospective	Food: meat	1987–1988	Winter
Dworkin, 2001 [30]	ReA, Reiter's	USA	Adults	63	Outbreak in community	Prospective	Food: meat	1994	Autumn/winter
Eastmond, 1983 [31]	ReA	Scotland	Adults	50	Outbreak in community	Prospective	Food: dairy	1981	Autumn
Hakansson, 1975 [32]	ReA	Sweden	n.r.	n.r.	Outbreak in community	Prospective	n.r.	1974	n.r.
Hannu, 2002 [33]	ReA	Finland	All ages	59	Outbreak in community	Prospective	Other	1999	Spring
Locht, 2002 [19]	ReA	Denmark	Adults	56	Outbreak in community	Prospective	Food: other	1999	Winter
Locht, 1993 [34]	ReA	Sweden	Adults	44	Outbreak in community	Prospective	Food: other	1990	Spring
Mattila, 1998 [35]	ReA, Reiter's	Finland	All ages	68	Outbreak in community	Prospective	Food: vegetable	1994	Spring
Mattila, 1994 [36]	ReA, Reiter's	Finland	All ages	62	Outbreak in community	Prospective	Food: vegetable	1992	Autumn
McColl, 2000 [37]	ReA	Australia	All ages	51	Outbreak in community	Prospective	Food: meat	1997	Autumn
McColl, 2000 [37]	ReA	Australia	All ages	47	Outbreak in community	Prospective	Food: meat	1997	Spring
McKendrick, 1994 [38]	IBS	UK	n.r.	66	Outbreak in community	Prospective	Food: other	Pre-1994	n.r.
Mearin, 2005 [39]	IBS	Spain	Adults	55·3	Outbreak in community	Prospective	Food: dairy	2002	Summer
Rudwaleit, 2001 [40]	ReA	Germany	Youth	n.r.	Outbreak in community	Prospective	Food: dairy	1998	Winter
Samuel, 1995 [41]	ReA	USA	n.r.	53	Outbreak in community	Prospective	Food: meat	1993	Summer
Thomson, 1992 [42]	ReA	n.r.	Adults	5	Outbreak in community	Prospective	Food: meat	Pre-1992	n.r.
Urfer, 2000 [43]	ReA, IBS	Switzerland	All ages	37	Outbreak in community	Prospective	Food: meat	1993	Autumn
Lee, 2005 [44]	ReA	Australia	All ages	48	Outbreak in community	Retrospective	Food: vegetable	1999	Summer/autumn
Thomson, 1995 [20]	ReA/Reiter's‡	n.r.	n.r.	n.r.	Outbreak in community	Retrospective	Food: other	1984	Autumn
Rohekar, 2008 [45]	ReA	Canada	Adults	71·3	Outbreak in community	Retrospective	Food: vegetable	2005	Autumn/winter
Thomson, 1994 [46]	ReA, Reiter's	n.r.	n.r.	n.r.	Outbreak in community	Retrospective	Food: meat	1990	Spring
Petersen, 1996 [47]	ReA	Denmark	All ages	49	Hospitalized cases	Prospective	n.a.	1991–1993	All
Saps, 2008 [48]	IBS	USA & Italy	All ages	48	Hospitalized cases	Prospective	n.a.	2006	Summer/autumn
Helms, 2006 [49]	GBS, IBD, IBS, HUS, ReA	Denmark	All ages	50	Surveillance	Retrospective	n.a.	1991–1999	All

GBS, Guillain–Barré syndrome; HUS, haemolytic uraemic syndrome; IBD, inflammatory bowel disease; IBS, irritable bowel syndrome; MFS, Miller Fisher syndrome; n.r., not reported; n.a., not applicable; ReA, reactive arthritis; UC, ulcerative colitis.

* Youth were individuals aged <18 years, adults were aged >18 years.

† Surveillance includes laboratory and notifiable disease registries, sporadic cases and other population surveillance.

‡ Study combined cases of ReA and Reiter's syndrome.

### Reactive arthritis

#### Study descriptions

Of the 32 studies investigating cases of NTS and chronic sequelae (Table 1), 25 provided outcomes for ReA. The 25 studies were from 11 countries, eight of which were European. Seventeen were based on outbreaks and of those that reported source (*n* = 16) all but one were foodborne and 44% (7/16) of those were attributed to contaminated meat. Of the seven surveillance studies, five were prospective in design. There was only a single study of hospitalized NTS cases. For all studies, follow-up times ranged from 28 to 1080 days (3 years) (Table 2).

**Table 2.** Outcome variables organized by chronic sequelae for studies relating to *Salmonella* published prior to July 2011

First author, year [ref.]	Species or serotype*	Sequelae negative prior to diagnosis with *Salmonella*?	Time from *Salmonella* diagnosis to evaluation for chronic sequelae (days)	Diagnosis of *Salmonella*†	Diagnosis of sequelae	Number of people with *Salmonella*	Number of people who developed sequelae	Outcome
**ReA**								
Arnedo-Pena, 2010 [28]	Hadar	All were disease negative	90	Probable	Disease status confirmed by specialist	155	13	8·4%
Arnedo-Pena, 2010 [28]	Hadar	All were disease negative	120	Probable	Self-reported based on validated scale	155	16	10·3%
Arnedo-Pena, 2010 [28]	Hadar	All were disease negative	150	Confirmed	Disease status confirmed by specialist	67	6	9·0%
Buxton, 2002 [27]	Typhimurium	All were disease negative	90	Confirmed	Disease status confirmed by specialist	61	4	6·6%
Buxton, 2002 [27]	Typhimurium	All were disease negative	120	Confirmed	Self-reported based on validated scale	66	17	25·8%
Doorduyn, 2008 [22]	n.r.	n.r.	1080	Confirmed	Self-reported	181	8	4·4%
Dworkin, 2001 [30]	Enteritidis	n.r.	30	Probable	Self-reported	217	63	29·0%
Eastmond, 1983 [31]	Typhimurium	n.r.	60	Confirmed	Medical records/physician‡	418	8	1·9%
Ekman, 2000 [23]	n.r.	All were disease negative	n.r.	Confirmed	Disease status confirmed by specialist	198	8	4·0%
Ekman, 2000 [23]	n.r.	All were disease negative	n.r.	Confirmed	Self-reported	198	13	6·6%
Hakansson, 1975 [32]	Typhimurium	n.r.	n.r.	Probable	n.r.	330	13	3·9%
Hannu, 2002 [33]	Typhimurium	All were disease negative	60	Confirmed	Disease status confirmed by specialist	63	5	7·9%
Helms, 2006 [49]	Combined	All were disease negative	365	Confirmed	Medical records/physician	27 894	87	0·3%
Helms, 2006 [49]	Enteritidis	All were disease negative	365	Confirmed	Medical records/physician	14 533	50	0·3%
Helms, 2006 [49]	Typhimurium	All were disease negative	365	Confirmed	Medical records/physician	7021	24	0·3%
Helms, 2006 [49]	Other	All were disease negative	365	Confirmed	Medical records/physician	6340	13	0·2%
Lee, 2005 [44]	Typhimurium	All were disease negative	90	Confirmed	Medical records/physician	261	38	14·6%
Lee, 2005 [44]	Typhimurium	All were disease negative	120	Confirmed	Medical records/physician	54	13	24·0%
Lee, 2005 [44]	Typhimurium	All were disease negative	150	Confirmed	Medical records/physician	207	25	12·1%
Locht, 2002 [19]	n.r.	No - excluded§	28	Probable	Self-reported	91	17	18·7%
Locht, 1993 [34]	Enteritidis	All were disease negative	30	Probable	Self-reported	108	17	15·7%
Locht, 1993 [34]	Enteritidis	All were disease negative	30	Confirmed	Self-reported	89	16	18·0%
Mattila, 1998 [35]	Bovismorbificans	All were disease negative	90	Confirmed	Disease status confirmed by specialist	191	22	11·5%
Mattila, 1994 [36]	n.r.	All were disease negative	150	Confirmed	Disease status confirmed by specialist	246	16	6·5%
McColl, 2000 [37]	Typhimurium	All were disease negative	90	Probable	Self-reported	312	13	4·2%
McColl, 2000 [37]	Typhimurium	All were disease negative	90	Probable	Self-reported	112	6	5·4%
Petersen, 1996 [47]	Enteritidis	n.r.	n.r.	Confirmed	Medical records/Physician	48	4	8·3%
Petersen, 1996 [47]	Typhimurium	n.r.	n.r.	Confirmed	Medical records/Physician	40	3	7·5%
Petersen, 1996 [47]	Other	n.r.	n.r.	Confirmed	Medical records/Physician	128	7	5·5%
Rohekar, 2008 [45]	Enteritidis	n.r.	n.r.	Confirmed	Self-reported	104	65	62·5%
Rudwaleit, 2001 [40]	Enteritidis	All were disease negative	120	Probable	Combination	286	0·5	0·2%
Samuel, 1995 [41]	n.r.	n.r.	196	Probable	Medical records/physician	321	23	7·2%
Schiellerup, 2008 [24]	Other	No – excluded	30	Confirmed	Self-reported based on validated scale	619	104	16·8%
Schiellerup, 2008 [24]	Typhimurium	No – excluded	30	Confirmed	Self-reported based on validated scale	193	29	15·0%
Schiellerup, 2008 [24]	Enteritidis	No – excluded	30	Confirmed	Self-reported based on validated scale	270	49	18·2%
Schiellerup, 2008 [24]	Other	No – excluded	30	Confirmed	Self-reported based on validated scale	156	26	16·7%
Ternhag, 2008 [21]	n.r.	n.r.	365	n.r.	Medical records/physician	34 664	27	0·08%
Thomson, 1992 [42]	Heidelberg	All were disease negative	30	Probable	Self-reported	73	6	8·2%
Thomson, 1994 [46]	Enteritidis	All were disease negative	60	Probable	Medical records/physician	29	8	27·6%
Townes, 2008 [25]	n.r.	All were disease negative	42	Confirmed	Combination	1356	17	1·3%
Urfer, 2000 [43]	Braenderup	n.r.	180	Probable	Self-reported	156	1	0·6%
**Reiter's**								
Dworkin, 2001 [30]	Enteritidis	n.r.	30	Probable	Meet study definition based on self-reported symptoms	217	6	2·7%
Doorduyn, 2008 [22]	n.r.	n.r.	1080	DNA based	Self-reported	193	0	0%
Mattila, 1998 [35]	Bovismorbificans	All were disease negative	90	Culture	Disease status confirmed by specialist	191	0	0%
Mattila, 1994 [36]	Enterica	All were disease negative	150	Culture	Disease status confirmed by specialist	246	0	0%
Thomson, 1994 [46]	Enteritidis	All were disease negative	60	Probable	Physician diagnosed	29	2	6·9%
**ReA/Reiter's combined**								
Thomson, 1995 [20]	*Typhimurium*	All were disease negative	90	Probable	Self-reported based on validated scale	423	27	6·4%
**GBS**								
Helms, 2006 [49]	Enteritidis	All were disease negative	365	Confirmed	Medical records/physician	14 533	1	0·01%
Helms, 2006 [49]	Typhimurium	All were disease negative	365	Confirmed	Medical records/physician	7021	1	0·01%
Helms, 2006 [49]	Other	All were disease negative	365	Confirmed	Medical records/physician	6340	2	0·03%
Doorduyn, 2008 [22]	n.r.	n.r.	1080	Confirmed	Self-reported	193	0	0·00%
**MFS**								
Doorduyn, 2008 [22]	n.r.	n.r.	1080	Confirmed	Self-reported	193	0	0·00%
**IBS**								
Ternhag, 2008 [21]	Spp.	n.r.	365	.	Medical records/physician	34 664	5	0·01%
Helms, 2006 [49]	Combined	All were disease negative	365	Confirmed	Medical records/physician	27 894	252	0·9%
Helms, 2006 [49]	Enteritidis	All were disease negative	365	Confirmed	Medical records/physician	14 533	125	0·9%
Helms, 2006 [49]	Typhimurium	All were disease negative	365	Confirmed	Medical records/physician	7021	67	1·0%
Helms, 2006 [49]	Other	All were disease negative	365	Confirmed	Medical records/physician	6340	60	1·0%
Doorduyn, 2008 [22]	n.r.	No - included§	1080	Confirmed	Self-reported	193	12	6·2%
Saps, 2008 [48]	n.r.	n.r.	180	Confirmed	Self-reported	24	9	37·5%
Urfer, 2000 [43]	Braenderup	n.r.	180	Probable	Self-reported	156	12	7·7%
McKendrick, 1994 [38]	Enteritidis	All were disease negative	365	Probable	Self-reported based on validated scale	38	12	31·6%
Mearin, 2005 [39]	Enteritidis	No – excluded	90	Probable	Self-reported based on validated scale	367	27	7·4%
Mearin, 2005 [39]	Enteritidis	No – excluded	180	Probable	Self-reported based on validated scale	341	37	10·9%
Mearin, 2005 [39]	Enteritidis	No – excluded	365	Probable	Self-reported based on validated scale	266	31	11·7%
**IBD**								
Helms, 2006 [49]	Combined	All were disease negative	365	Confirmed	Medical records/physician	27 894	125	0·5%
Helms, 2006 [49]	Enteritidis	All were disease negative	365	Confirmed	Medical records/physician	14 533	50	0·3%
Helms, 2006 [49]	Typhimurium	All were disease negative	365	Confirmed	Medical records/physician	7021	47	0·7%
Helms, 2006 [49]	Other	All were disease negative	365	Confirmed	Medical records/physician	6340	28	0·4%
Doorduyn, 2008 [22]	n.r.	No – included	1080	Confirmed	Self-reported disease status	193	12	6·2%
**Crohn's**								
Ternhag, 2008 [21]	spp.	n.r.	365	n.r.	Medical records/physician	34 664	14	0·04%
Jess, 2011 [26]	Combined	All were disease negative	up to 16 years	Confirmed	Medical records/physician	41 628	78	0·2%
**UC**								
Ternhag, 2008 [21]	spp.	n.r.	365	n.r.	Medical records/physician	34 664	29	0·08%
Jess, 2011 [26]	Combined	All were disease negative	up to 16 years	Confirmed	Medical records/physician	41 628	264	0·6%
Cowden,1989 [29]	Typhimurium	n.r.	n.r.	Confirmed	n.r.	85	1	1·2%

GBS, Guillain–Barré syndrome; IBD, inflammatory bowel disease; IBS, irritable bowel syndrome; MFS, Miller Fisher syndrome; n.r., not reported; ReA, reactive arthritis; UC, ulcerative colitis.

* Combined = study combined non-typhoid strains; Other = non-typhoid strains other than Enteritidis and Typhimurium.

† Confirmed for *Salmonella* included those confirmed by culture, DNA-based tests and serology. Probable includes those identified as a case of non-typhoidal salmonellosis based on clinical case definition from study.

‡ Medical records/physician included those hospitalized for sequelae or diagnosed by a physician.

§ No – excluded: cases of non-typhoidal salmonellosis with previous medical history of related sequelae were excluded from analysis. No – included: cases of non-typhoidal salmonellosis with previous medical history of related sequelae were not excluded from analysis.

#### Outcomes

**Fig. 2.**
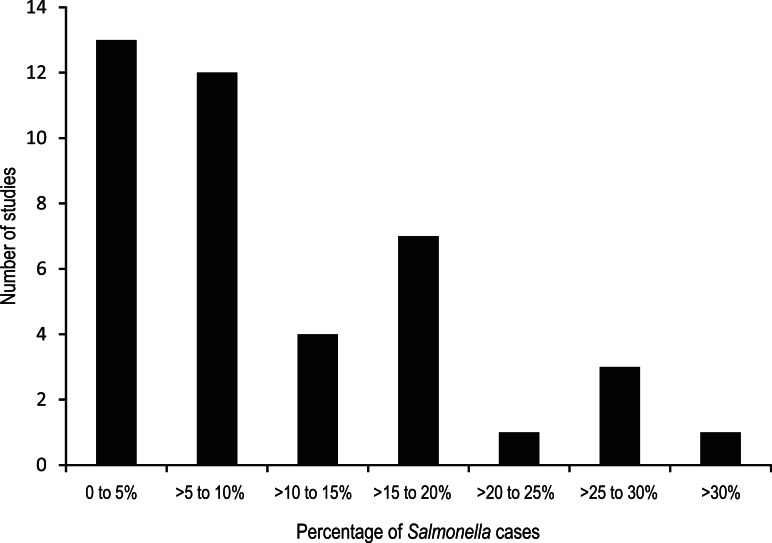
Distribution of proportions of non-typhoidal salmonellosis cases that developed reactive arthritis from 32 studies published prior to July 2011.

There were 42 outcomes (Table 2), as 13 studies provided multiple outcomes. In all but one of the surveillance-based studies, cases were laboratory confirmed for *Salmonella.* The number of NTS cases ranged from 61 to 34 664 persons, with the probability of a case developing ReA ranging from 0·08% to 25·8%. For outbreak studies, confirmed NTS cases ranged from 63 to 418 cases with the proportion of cases developing ReA varying from 1·9% to 62·5%. The case numbers for probable NTS cases from outbreaks ranged from 29 to 330 persons with 0·2–29·0% of cases developing ReA. Overall, most studies reported estimates of <10% of NTS cases developing ReA (Fig. 2).

#### Assessment of factors associated with internal or external validity

The majority (84%, 21/25) of studies reported the time from *Salmonella* diagnosis to diagnosis for ReA. Nine studies (36%) did not report whether or not individuals were negative for ReA prior to illness with *Salmonella*. Age range and gender distribution of NTS cases were not reported in 24% (6/25) and 16% (4/25) of studies, respectively. Data source (surveillance *vs*. hospitalized cases *vs*. outbreak), study directionality (retrospective *vs*. prospective), and method for sequelae diagnosis were reported in all studies. The method for how *Salmonella* was diagnosed was not reported in a single study. The specific diagnostic criteria used in sequelae diagnosis were not reported in six studies.

#### Meta-analysis/meta-regression

**Fig. 3.**
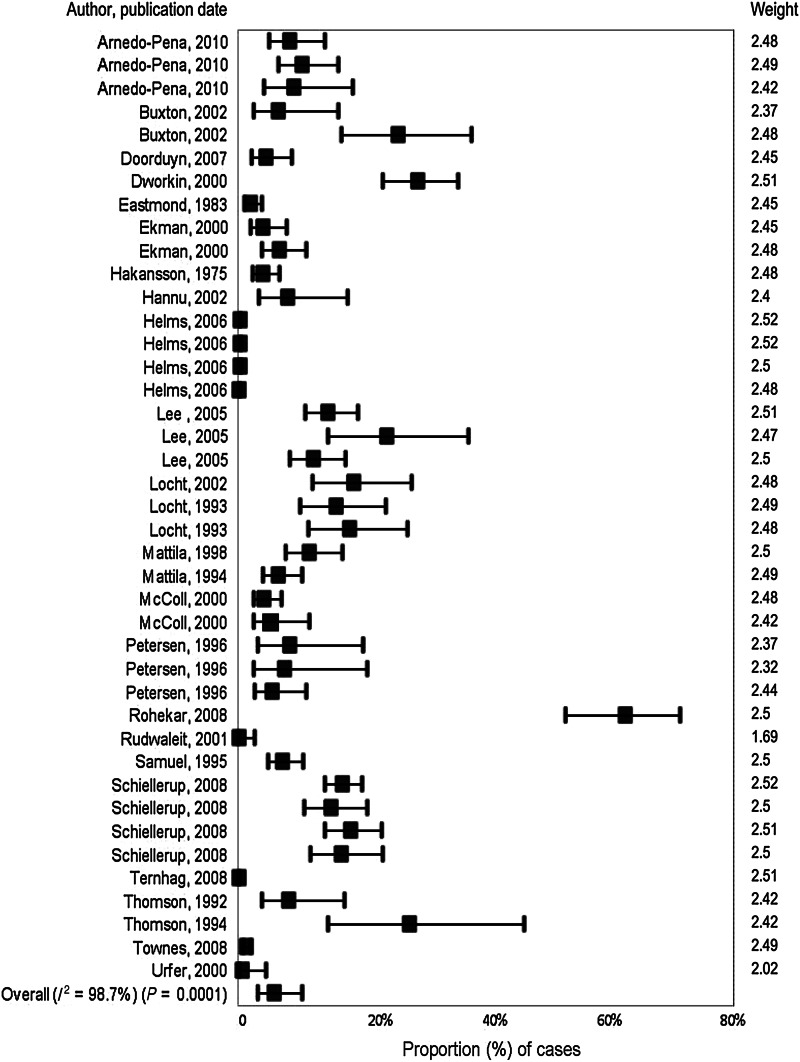
Forest plot from meta-analysis of cases of non-typhoidal salmonellosis and reactive arthritis for studies published prior to July 2011.

A total of 42 outcomes were included in the meta-analysis for *Salmonella* and ReA. The overall estimate of the proportion of NTS cases that developed ReA was 5·8% [95% confidence interval (CI) 3·2–10·3, *I*^2^ = 98·7%] (Fig. 3). Because of the high heterogeneity, meta-regression was used to explore potential sources.

Variables significantly contributing to heterogeneity in univariable analysis were group size (*P* < 0·0001), country (*P* = 0·046), source of data (*P* = 0·014), follow-up time (*P* < 0·001) and sequelae diagnosis (*P* = 0·013) (Table 3). In the multivariable analysis, only group size remained significant (*P* = 0·037) (Table 4). However, group size is not a factor that could directly impact the outcome, and there was considerable correlation between group size and the other, more biologically plausible, variables considered in the univariable analysis (data not shown). Therefore, the multivariable model was re-run excluding the group size variable. Both follow-up time (*P* = 0·001) and method of sequelae diagnosis (*P* = 0·045) remained significant.

**Table 3.** Summary of variables explored in meta-analysis by sequelae

Sequelae	Study/population variables explored with univariable analysis	Study/population variables explored with multivariable analysis
Reactive arthritis	Time from *Salmonella* diagnosis to evaluation for chronic sequelae (follow-up time) *Salmonella* species or serotype Diagnosis of *Salmonella* (probable *vs*. confirmed) Method of sequelae diagnosis Source of data Study directionality Season Country Group size	Group size Country Source of data Follow-up time Diagnosis of sequelae
Irritable bowel syndrome	Time from *Salmonella* diagnosis to evaluation for chronic sequelae (follow-up time) *Salmonella* species or serotype Diagnosis of *Salmonella* (probable *vs*. confirmed) Method of sequelae diagnosis Source of data Study directionality Season Country Group size	Group size Country Source of data Study directionality Method of sequelae diagnosis

**Table 4.** Subgroup meta-analyses by group size, follow-up time and sequelae diagnosis for *Salmonella* and reactive arthritis

Variable	Summary estimate	(95% CI)	*I*^2^	Number of observations
Overall estimate	5·8%	(3·2–10·3)	98·7%	42
Group size				
Small	14·2%	(10·3–19·4)	62·7%	11
Medium	9·0%	(6·4–12·7)	93·6%	24
Large	0·5%	(0·2–1·2)	92·8%	3
Extra large	0·2%	(0·09–0·5)	95·7%	3
Follow-up time, days				
<90	12·0%	(7·7–18·2)	94%	13
90 days to <365	9·0%	(6·5–12·3)	80·6%	15
⩾365	0·4%	(0·2–0·7)	95·4%	6
Sequelae diagnosis				
Specialist	7·7%	(5·9–10·1)	27·1%	7
Physician diagnosed/medical records	2·6%	(0·9–7·3)	98·9%	14
Self-reported based on validated scale	16·6%	(14·0–19·7)	44·5%	6
Self-reported	11·1%	(5·5–21·1)	95·3	11

CI, Confidence interval.

Subgroup meta-analysis estimated the proportion of NTS cases that developed ReA as 12·0% (95% CI 7·7–18·2, *I*^2^ = 94%) in studies where follow-up occurred within 90 days compared to 0·4% (95% CI 0·2–0·7, *I*^2^ = 95·40%) in those that occurred ⩾1 year after *Salmonella* infection (Table 4). In a separate subgroup meta-analysis, the method of diagnosis was significant with rates of ReA estimated as 7·7% (95% CI 5·9–10·1, *I*^2^ = 27·1%) in cases confirmed by a specialist, 2·6% (95% CI 0·9–7·3, *I*^2^ = 98·9%) in cases identified by medical records, 16·6% (95% CI 14·0–19·7, *I*^2^ = 44·5%) in those that were self-reported using a validated scale and 11·1% (95% CI 5·5–21·1, *I*^2^ = 95·3%) in self-reported cases.

### Reiter's syndrome

RS is related to ReA and is diagnosed based on a triad of symptoms; arthritis in combination with conjunctivitis and urethritis [6]. Five studies, each with one outcome, were examined for *Salmonella* and RS. Four studies were based on outbreaks and the proportion of RS ranged from 0% to 6·90%. One publication presented ReA and RS in combination, with the proportion of cases that developed either sequelae equal to 6·6%. Due to insufficient data, a summary proportion was not calculated.

### ReA and RS combined

The study by Thomson *et al*. [20] included cases of RS in their definition for ReA. Due to the inconsistent case definition, these results were not included in the meta-analysis for ReA (Table 2).

### Irritable bowel syndrome

#### Study descriptions

Seven studies provided information on *Salmonella* and IBS (Table 1). The studies were from seven different European countries, with one of the studies presenting both US and Italian data. Design types included outbreak (3/7) and surveillance (3/7) studies with one study of hospitalized cases. The majority (71%, 5/7) were prospective and follow-up times ranged from 3 months (90 days) to 3 years (1080 days) (Table 2).

#### Outcomes

There were 12 outcomes, as two studies [39, 49] reported multiple outcomes (Table 2). For surveillance-based studies, the number of NTS cases varied from 193 and 34 664. All were confirmed NTS cases and the proportion of NTS cases developing IBS ranged from 0·01% to 6·2%. Outbreak studies ranged from 38 to 367 cases of NTS of which none were confirmed for *Salmonella* and IBS occurred in 7·4% to 31·6% of cases.

#### Assessment of factors associated with internal or external validity

Data on age range of the NTS cases was not reported in any study and two studies did not report information on gender distribution of NTS cases. Study directionality and source of data were reported in all studies. The method of diagnosis for *Salmonella* was not reported in any publication. Follow-up time was reported in all studies but three studies did not report on whether all *Salmonella* cases were disease negative for IBS prior to the onset of *Salmonella*. All studies reported the method of diagnosing IBS (physician *vs*. self-reported); however, the specific diagnostic criteria used were not reported in four of the studies.

#### Meta-analysis/meta-regression

**Fig. 4.**
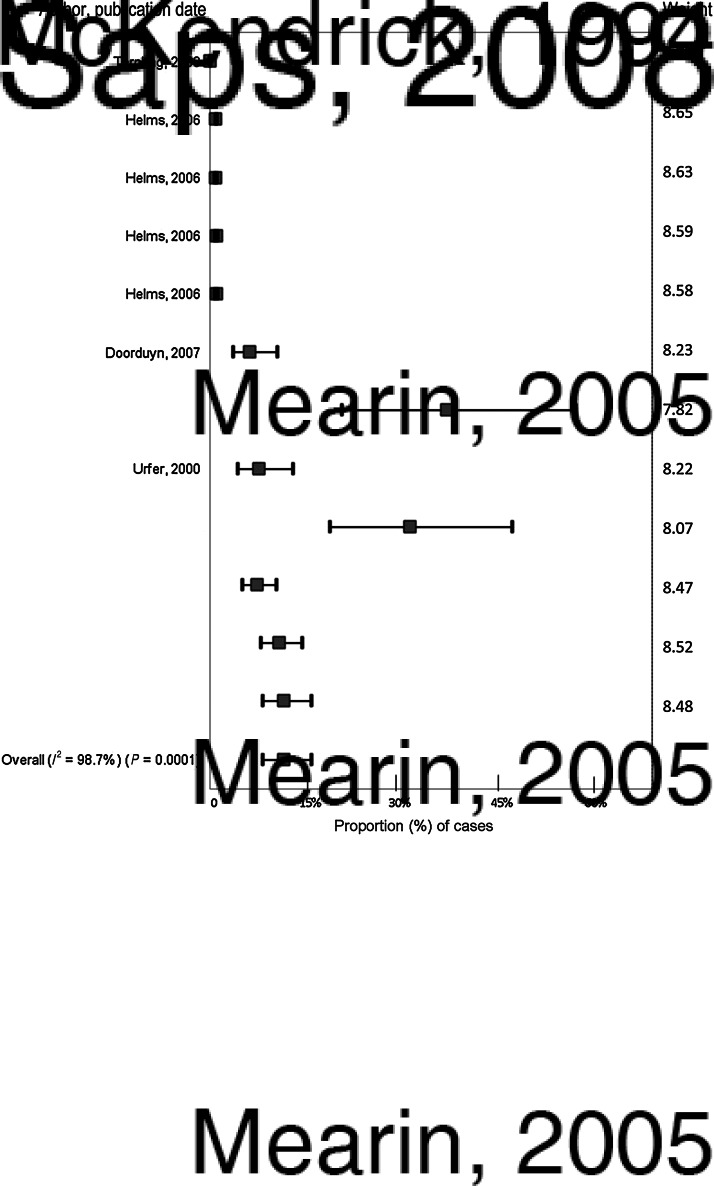
Forest plot from meta-analysis of cases of non-typhoidal salmonellosis and IBS from studies published prior to July 2011.

Twelve outcomes were included in the analysis. The estimate for the proportion of NTS cases that developed IBS was 3·3% (95% CI 1·6–6·6, *I*^2^ = 98·7) (Fig. 4). In univariable analyses, group size (*P* < 0·001), country (*P* = 0·006), source of data (*P* = 0·014), study directionality (*P* = 0·001), method of IBS diagnosis (*P* = 0·008) significantly contributed to the heterogeneity in the data (Table 3). Age range of NTS cases (*P* = 0·057) was close to the pre-specified significant cut-point. Due to the potential biological significance and implications in BOD measures it was included in the model. In a multivariable analysis, all variables remained significant except for group size. Due to the limited number of studies, subgroup meta-analyses of these factors was not possible.

### IBD including Crohn's disease and UC

IBD includes Crohn's disease and UC, which share similar symptomology and pathogenesis but are considered separate diseases [17]. Two studies reported a total of five different outcomes for *Salmonella* and IBD (Table 2). All were surveillance studies of confirmed cases of NTS with the proportion of NTS cases developing IBD ranging from 0·3% to 6·2%. Two studies reported results for Crohn's disease. Both were large retrospective studies where the occurrence of Crohn's disease was 0·04% and 0·2%. Three studies reported on the proportion of NTS cases that developed UC. Estimates ranged from 0·08% in culture-confirmed cases of NTS to 1·2% in those not reporting how *Salmonella* was diagnosed. Due to insufficient data, a summary proportion was not calculated.

### Guillain–Barré syndrome

Two studies provided four outcomes for *Salmonella* and GBS. Both were surveillance studies with GBS occurring in 0% and 0·03% of confirmed NTS cases (Table 2). Due to insufficient data, a summary proportion was not calculated.

### Miller Fisher syndrome

MFS is an uncommon variant of GBS, which is characterized by ophthalmoplegia, ataxia and areflexia [18]. One prospective surveillance study from The Netherlands reported results for MFS. None of the 193 NTS cases developed MFS (Table 2). As there was only a single outcome, meta-analysis was not performed.

### Haemolytic uraemic syndrome

Two studies reported a total of five different outcomes for *Salmonella* and HUS (Table 2). Both were large retrospective surveillance studies with HUS reported in 0·003% and 0·016% of cases. Due to insufficient data, a summary proportion was not calculated.

## DISCUSSION

In this study, the results of a systematic literature search and meta-analysis were used to estimate the proportion of NTS cases that developed ReA, and IBS. A range of sequelae was considered in this review; although not all of these sequelae are commonly associated with *Salmonella* they were included as they have been associated with other foodborne pathogens. There were insufficient data to calculate summary proportions for the other sequelae considered in this review. A comprehensive literature search of multiple databases was conducted making it probable that this review captured the majority of research available on the proportion of NTS cases that develop these chronic sequelae. Contributing to the limited number of studies on chronic sequelae could be the issue of under-reporting and under-diagnosis of *Salmonella* [8], as links between infection and sequelae development would not be evaluated on unidentified cases. In addition, when specific sequelae were not reported, it was not possible to determine whether they were not evaluated or whether they were evaluated but not identified in any of the cases.

Although data were limited for some of the sequelae of interest, estimates were calculated for ReA and IBS. Similar to results reported for *Campylobacter* [12], group size and follow-up time were significantly associated with the proportion of NTS cases that developed ReA. The lower proportion of ReA reported in larger study sizes could be due to differences between study types in case definition, case ascertainment, or intensity of follow-up. Stratifying estimates within study type (e.g. large population studies and outbreak studies) would potentially result in more consistent estimates; however, there were a small number of studies for each design type.

Although all our results indicate that the proportion of cases developing ReA was higher when evaluated within 90 days, evidence of arthritis lasting over 5 years post-infection exists [20]. The higher proportions observed with shorter follow-up time could be attributed to whether studies were capturing incident or prevalent cases and three scenarios could potentially explain this finding; the publications captured a high number of incident cases of short disease duration (within 90 days), others could be capturing remaining prevalent cases that were incident cases within first 3 months and remained symptomatic, while others could be capturing incident cases that developed after 90 days or a combination of these. From a BOD perspective, clarification surrounding disease progression and duration is important considering the relative impact on health systems of a high number of cases of ReA of short duration *vs.* a low number of chronic cases. As these two situations may require different resources and incur different costs, there is the potential need to separate them when calculating costs/resource requirements. Future studies with sequential sampling of the same individuals over time may shed some light on the duration of ReA. In addition, reporting on the specifics of the questions used during follow-up could add clarity as to whether the study reported incident or prevalent cases.

The method of diagnosis for the sequelae was also a significant contributor to heterogeneity for cases of NTS and ReA. There was a large range of diagnostic criteria used within the physician/specialist confirmed studies. The lack of a clear definition for ReA [6] is problematic as it prevents meaningful comparison between studies. Moreover, the higher proportion of ReA cases for studies based on self-reported results indicate that personal perception may play a role in disease status. The wording and timing of questions investigating ReA could influence the differences seen in diagnostic method but with the limited details published in most studies it was not possible to investigate these effects further.

For IBS, several variables were significantly associated with heterogeneity in univariable analysis. However, it is difficult to interpret the results due to the low number of studies, and inter-relatedness between variables. Thus, further research is needed to accurately estimate the proportion developing IBS and the factors associated with this.

For ReA and IBS, heterogeneity remained high after subgroup analysis, suggesting that additional sources of heterogeneity are present. Some of the factors that were evaluated, such as age, were inconsistently reported in the primary studies, limiting a comprehensive evaluation of their impact on heterogeneity. Analyses of sources of heterogeneity, and the interpretation of those analyses was complicated by the inter-relatedness of many of the variables and the small sample size available for evaluating potential confounding or interaction between variables. For instance, surveillance studies also tended to be retrospective and generally also had the larger samples sizes. Additional sources of heterogeneity could be related to host, pathogen and environmental factors not evaluated in this study. For example the race, gender or immune status of sequelae cases, the dose of pathogen received, severity of acute illness and virulence of the *Salmonella* strain are all potential sources of heterogeneity that were not investigated. For many of these factors, the information is not easily ascertained from the published literature.

A number of the variables evaluated were not significantly associated with heterogeneity. However, given the small number of studies and low power to detect significant differences, non-significant results should be interpreted with caution.

There were several additional limitations to this study. A relatively large number of potentially relevant publications were not in English and resources were not available to translate these reports. It is not known the extent to which this would impact the results. Future studies should consider acquiring resources for translation or estimating country-specific estimates, if sufficient data are available. Categorization of group size and follow up time introduced the potential for bias as the categories were determined *post-hoc*. The assumption of independence of outcomes for the meta-analysis was not met as multiple outcomes were used from some studies. This in combination with the large amount of unidentified heterogeneity indicates the summary estimates reported in this study must be interpreted with caution.

Reporting of key features was not consistent in publications. This made interpretation of the results difficult. In particular, non-reporting of results by age meant that estimates could not be calculated within age group, limiting the usefulness of these results in burden of illness studies where measures such as DALYs calculate burden based on frequency of illness within age groups. Missing data also decreased power to detect significant sources of heterogeneity. Although the review included a range of study designs, guidelines for reporting research studies are available for many study designs, including observational studies (the STROBE statement) [50], surveys [51], and outbreaks (the ORION statement) [52]. Comprehensive and transparent reporting of research execution and results is essential to the use of those results for secondary purposes. Over time, use of guidelines could result in a more consistent body of data to inform burden of illness studies.

## CONCLUSION

Estimates for the proportion of NTS cases developing ReA and IBS were calculated. However, extremely high heterogeneity in the estimates, likely due to differences in methodology between studies, limits the usefulness of these estimates. For more accurate estimates to be developed, consistent diagnostic approaches and case definitions need to be implemented and reported in future research.
